# *N*′^1^,*N*′^3^-Dimethyl-*N*′^1^,*N*′^3^-bis(phenylcarbonothioyl) Propanedihydrazide (Elesclomol) Selectively Kills Cisplatin Resistant Lung Cancer Cells through Reactive Oxygen Species (ROS)

**DOI:** 10.3390/cancers1010023

**Published:** 2009-12-15

**Authors:** Medhi Wangpaichitr, Chunjing Wu, Min You, Johnathan C. Maher, Vy Dinh, Lynn G. Feun, Niramol Savaraj

**Affiliations:** 1 V.A. Medical Center, Hematology/Oncology, Miami, FL 33125, USA; E-Mails: mwangpaichitr@med.miami.edu (M.W.); chunjingwu@hotmail.com (C.W.); vdinh@med.miami.edu (V.D.); 2 Sylvester Cancer Center, School of Medicine, University of Miami, Miami, FL, 33136, USA; E-Mails: jmaher@med.miami.edu (J.C.M.); myou@med.miami.edu (M.Y.); lfeun@med.miami.edu (L.G.F.)

**Keywords:** lung cancer, drug resistant, cisplatin, ROS, elesclomol

## Abstract

Cisplatin is an important chemotherapeutic agent in lung cancer treatment. The mechanism of drug resistance to cisplatin is complex and historically has been difficult to overcome. We report here that cisplatin resistant lung cancer cell lines possess high basal levels of reactive oxygen species (ROS) when compared to normal cells and their parental cell counterparts. These resistant cells also have low thioredoxin (TRX) levels which may be one of the contributory factors to high ROS. *N*′^1^,*N*′^3^-dimethyl-*N*′^1^,*N*′^3^-bis(phenylcarbonothioyl) propanedihydrazide (elesclomol), an agent known to increase ROS is selectively toxic to cisplatin-resistant cells, while sparing normal cells and the parental counterpart. The cytotoxic effect of elesclomol in resistant cells is accompanied by further decreases in TRX and glutathione (GSH) antioxidant systems, while opposite results were found in parental cells. The ID_50_ of elesclomol in cisplatin-resistant cells ranged from 5–10 nM, which is well within clinically achievable ranges. *N*-Acetylcysteine (NAC), which is known to neutralize ROS, can abolish the cytotoxic effect of elesclomol, suggesting that the cytotoxic effect results from increased ROS. Overall, our data suggest that elesclomol selectively kills cisplatin-resistant tumor cells through increased ROS. This agent may hold potential to overcome cisplatin resistance and should be further explored to treat patients who have failed cisplatin therapy.

## 1. Introduction

Cisplatin resistance often involves multiple mechanisms including drug transport, inactivation of drug, and cell type dependent DNA repair, thus making resistance to cisplatin difficult to overcome. One of the known pharmacologic actions of cisplatin is disruption of the redox system through inhibition of thioredoxin reductase (TrxR) [1,2]. TrxR is an important antioxidant system, similar to glutathione reductase (GshR), that converts H_2_O_2_ to water and O_2_ [3]. Thus, inhibition of TRX by cisplatin leads to increased ROS resulting in further damage of DNA and subsequent cell death [4,5].

It is well accepted that ROS can have either prosurvival or prodeath effects, depending on the amount and the effectiveness of the antioxidant system. In this report, we have found that cisplatin resistant cell lines harbor high basal levels of ROS which could be exploited as a therapeutic target. *N*′^1^,*N*′^3^-dimethyl-*N*′^1^,*N*′^3^-bis(phenylcarbonothioyl)propanedihydrazide (elesclomol) is a novel agent derived from a phenotypic screen for small molecules with potent proapoptotic activity through the induction of oxidative stress [6]. Treatment with elesclomol results in rapid generation of ROS and the induction of genes transcription that are responsible for oxidative stress [6]. In this study, we evaluate the antitumor effect of this agent in a panel of small cell lung cancer (SCLC) and non small cell lung cancer (NSCLC) cisplatin resistant cell lines.

## 2. Results and Discussion

### 2.1. Cisplatin Resistant Cell Lines Lead to Increased ROS Levels

**Figure 1:**
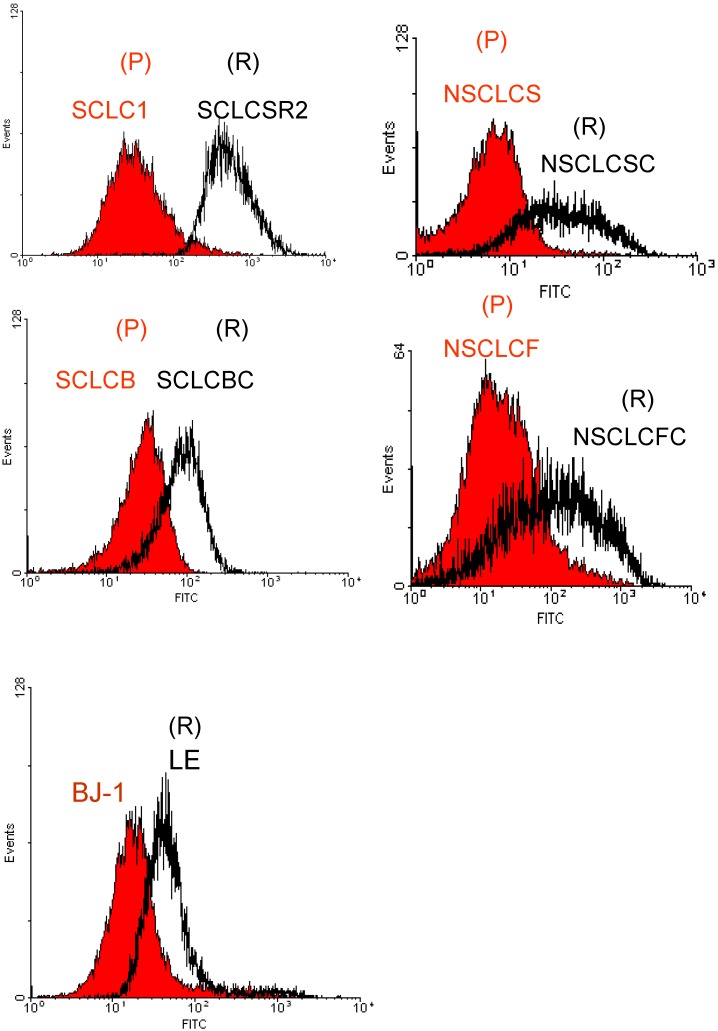
Flow cytometry analysis of ROS in various lung cancer cell lines detected by DCF-DA probe. The cisplatin resistant variants (R) are shown to possess higher basal levels of ROS when compared to parental cells counterpart (P). Each histogram is representative of three experiments (average P < 0.03, parental *vs.* resistant). BJ-1 was used as control.

We have studied two pairs of small cell lung cancer (SCLC), two pairs of non small cell lung cancer (NSCLC) and their cisplatin resistant counterparts as well as normal fibroblast cell line, BJ-1 (see Experimental section for ID_50_ response). Our results showed that ROS is increased in all cisplatin resistant cell lines when compared to their parental counterpart cells. In addition, we also evaluated the level of ROS in primary tumor cell cultures from a patient who failed cisplatin (cell line LE). Since no parental cells were available for comparison with LE cell line, normal cells BJ-1 were used as control. Similarly to cisplatin-resistant cell lines, primary tumor cells were also found to have high levels of ROS as compared with BJ-1 (Figure 1).

**Figure 2:**
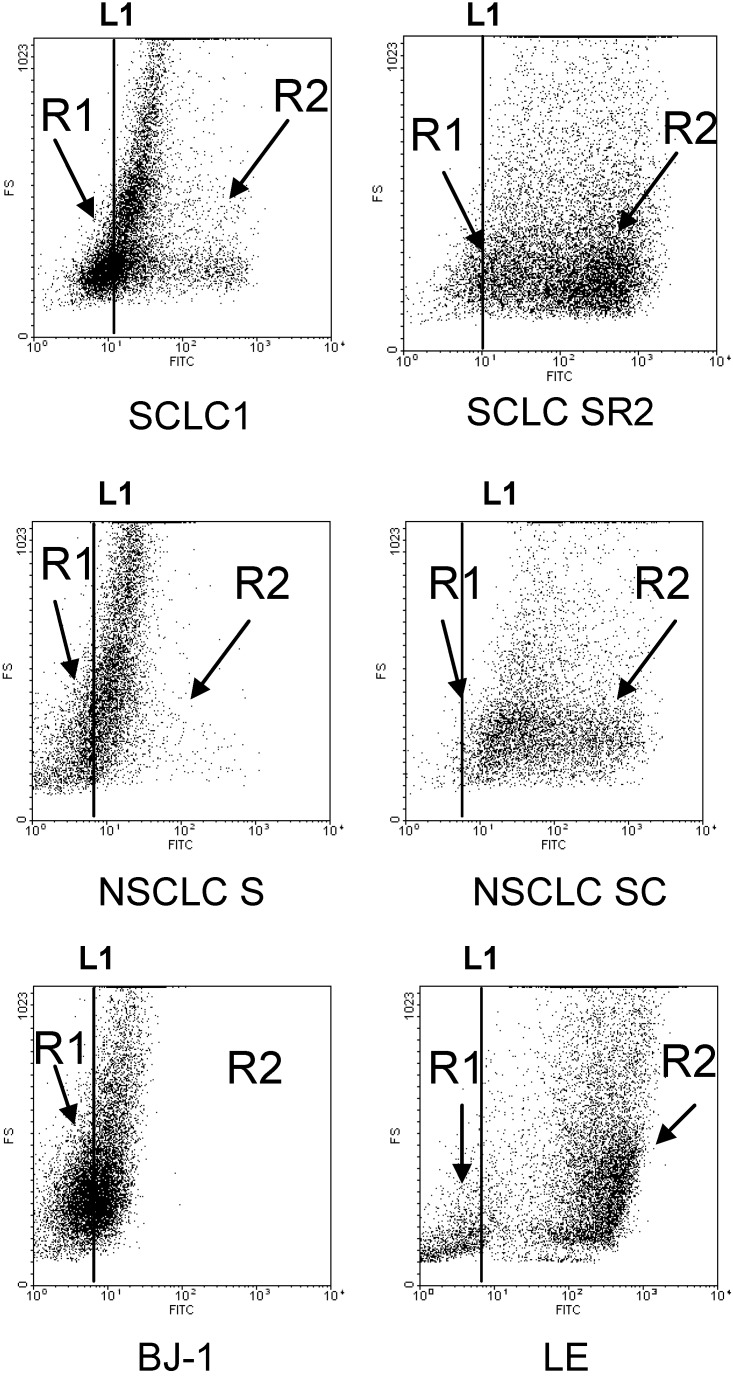
Flow cytometry analysis of H_2_O_2_ in lung cancer cell lines detected by APFB probe indicated that there are 2 populations of cells: R1 (low H_2_O_2_ production) and R2 (high H_2_O_2_ production). Resistant cells SCLCSR2, NSCLCSC, and LE have more R2 population than cisplatin sensitive cells and BJ-1. Each dot plot is representative of three experiments. All cisplatin resistant cell lines have more R2 than R1 population with an average *P* < 0.005. Line L1 is arbitrarily set as the average H_2_O_2_ production in the parental cells counterpart.

To further confirm that cisplatin resistant cell lines possess high basal ROS levels, we assayed H_2_O_2_ levels in the cell line pairs and the cisplatin-resistant primary culture using H_2_O_2_ specific APFB probe. The results are shown in Figure 2. All cisplatin-resistant cell lines clearly expressed high levels of H_2_O_2_ when compared to their parental cell counterparts. Again, normal BJ-1 cells were used for comparison with the primary cisplatin-resistant cells. Our data strongly indicate that cisplatin resistant cell lines possess intrinsically high basal ROS levels than their parental counterparts and normal cells.

### 2.2. Growth Inhibitory Effect of Elesclomol on Cisplatin Resistant Cell Lines

**Figure 3:**
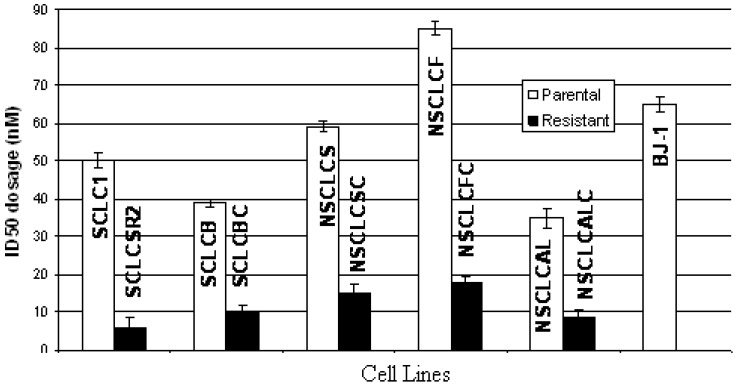
ID_50_ dosage of elesclomol in parental and cisplatin resistant lung cancer cell lines. (SD of three experiments, average p values < 0.005; parental *vs.* resistant). BJ-1 was used as control.

Since cisplatin resistant cell lines expressed substantially high level of ROS, we hypothesized that compounds that generate ROS may increase the ROS levels beyond the threshold of tolerability in cisplatin-resistant cells resulting in cell death while sparing normal cells. To confirm this concept, we have treated both parental and resistant cells with elesclomol, a compound known to increase ROS for 48 h [6]. The data are shown in Figure 3.

**Figure 4:**
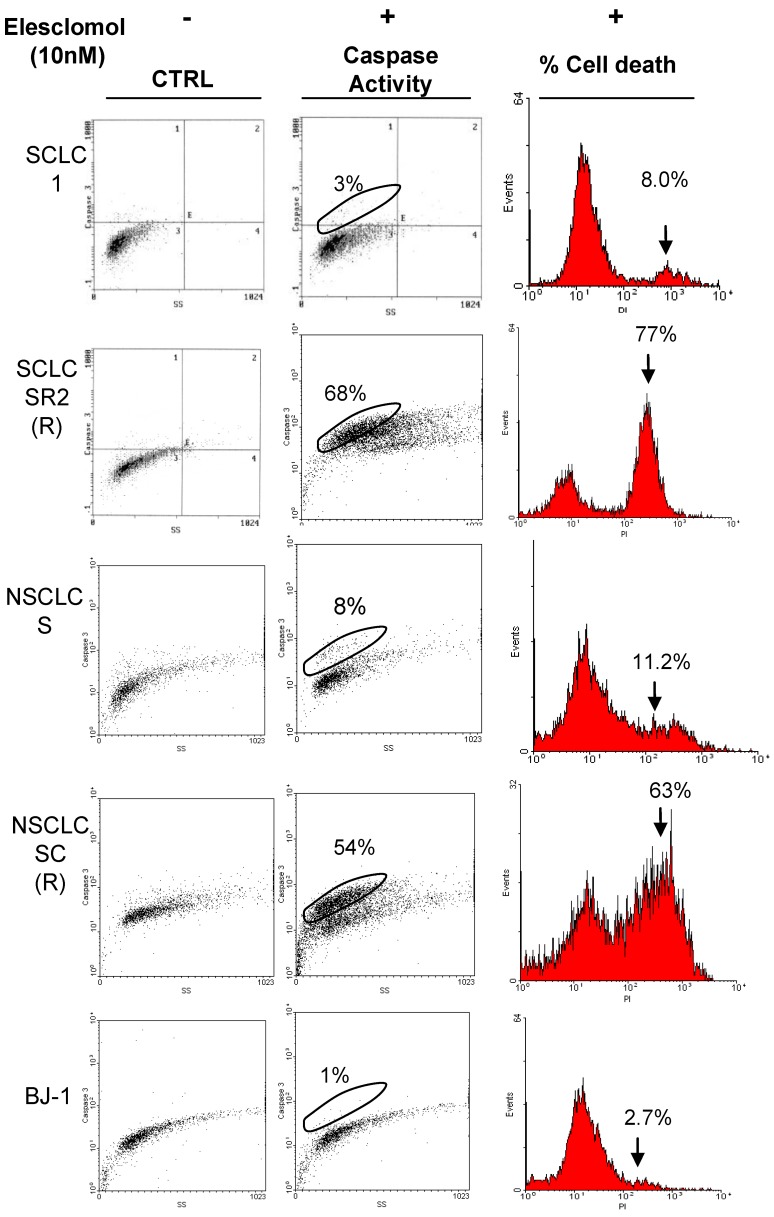
Elesclomol greatly stimulates caspase activity and cell death in cisplatin resistant cell line. Representative groups of SCLC and NSCLC were treated with 10 nM of elesclomol for 48 h and followed by caspase activity probe FMK-Fluoroscein. The percentage of caspase activation is correlated with percentage of cell death which was detected by PI staining. Elesclomol also has no effect against normal fibroblast BJ-1. Each dot plots and histograms are representative of two experiments.

Our results clearly demonstrated that elesclomol selectively kills cisplatin resistant cells. The growth inhibitory dosages (ID_50_) of elesclomol in cisplatin resistant cell lines are 4–10 times lower than their parental cells counterpart. We have also measured cell death and caspase activity by flow cytometry. Cell death resulting from the addition of 10 nM of elesclomol ranged from 60%–80% in cisplatin resistant cells and only 5%–10% in parental cells using the same dosage and only 2% cell death in BJ-1. A representative assay from each cell pair is shown in Figure 4.

### 2.3. Elesclomol Increased Levels of ROS and H_2_O_2_ in Cisplatin Resistant Cell Lines

**Figure 5:**
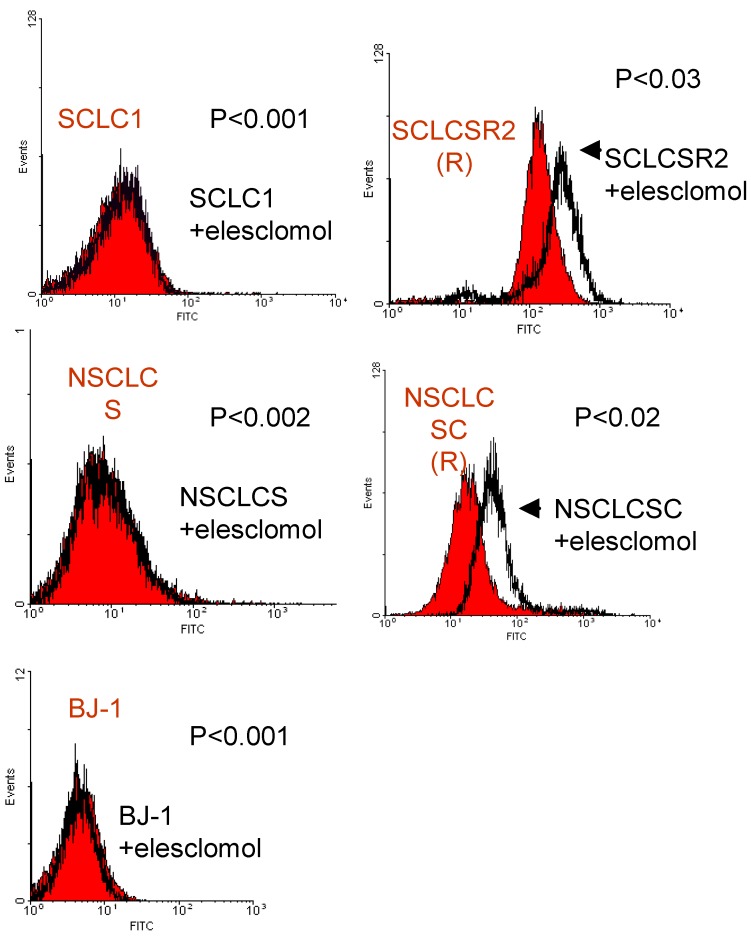
Flow cytometry analysis of ROS in a pair of SCLC and NSCLC cell lines detected by DCF-DA probe. 10 nM of elesclomol for 24 h greatly induced intracellular ROS levels in the resistant (R) cell lines SCLCSR2 and NSCLCSC whereas in parental cell lines (SCLC1 and NSCLCS) and BJ-1, no significant increase of ROS was seen. Each histogram is representative of three experiments; (nontreated *vs.* treated).

To determine whether ROS is increased after treatment with elesclomol, we have treated these two pairs of SCLC, NSCLC, and BJ-1 cells with 10 nM of elesclomol for 24h. Despite the short period of exposure time, the ROS levels are increased more in resistant cells after elesclomol treatment while no significant change occurred in parental cells or normal cells, BJ-1 (Figure 5).

**Figure 6:**
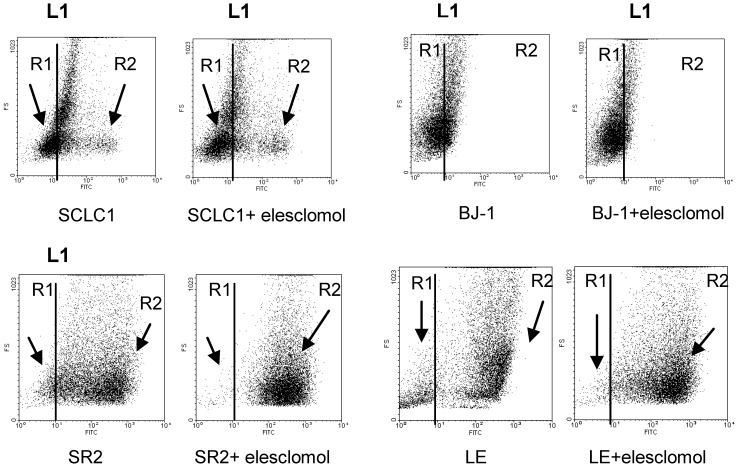
Flow cytometry analysis of H_2_O_2_ in lung cancer cell lines detected by APFB probe indicated that there are 2 populations of cell: R1 (low H_2_O_2_) and R2 (high H_2_O_2_). The levels of H_2_O_2_ decreased in parental lines (SCLC1) and normal cells (BJ-1) when treated with 10 nM of elesclomol for 24 h as indicated by the population of cells moved to the left of line L1 (more R1 population). SR2 and LE have more R2 population than cisplatin sensitive cells. Treatment with elesclomol increased the level of H_2_O_2_ production in the resistant cell line SR2 as well as in primary culture, LE as indicated by the shifting of R1 population to R2. Each dot plot is representative of three experiments (average P < 0.005; R1 *vs.* R2).

To further confirm this finding, we have also assayed H_2_O_2_ production pre and post elesclomol treatment. The results are shown in Figure 6. Similar to ROS, H_2_O_2_ production also increased after treatment with elesclomol in cisplatin resistant cells. In contrast, H_2_O_2_ production is decreased in parental cells and normal cells when treated with this ROS inducing agent. These results are interesting since parental cells and normal cells may have the ability to effectively turn on the antioxidant system to eliminate H_2_O_2_ accumulation.

**Figure 7:**
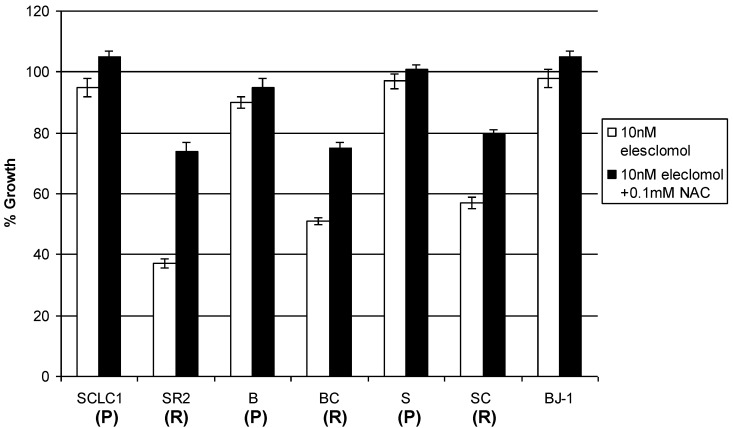
0.1 mM of *N*-acetylcysteine (NAC) is able to reverse the growth inhibitory effect of elesclomol in cisplatin resistant cell lines (R).

**Figure 8:**
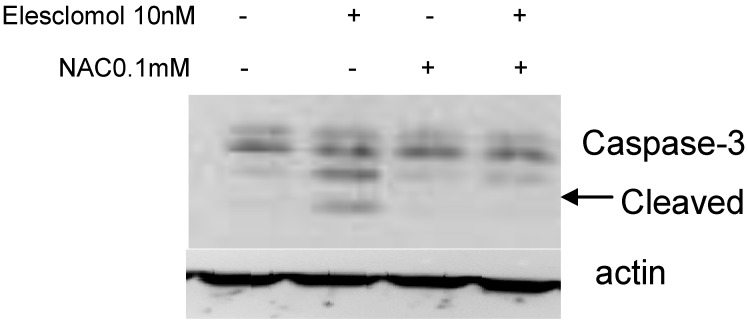
Immunoblot analysis of cleaved caspase-3 on SCLCSR2. Treatment with 10 nM of elesclomol for 72 h induced caspase 3 cleavages (arrow) which can be reversed with cotreatment of 0.1 mM of NAC (72 h).

To further determine whether increasing ROS leads to cell death, we have cotreated a known antioxidant agent *N*-acetylcysteine (NAC) with elesclomol and found that 0.1 mM of NAC can reverse the growth inhibitory effect of elesclomol as well as caspase-3 cleavage in cisplatin resistant cells (Figure 7 and Figure 8). Thus, our data further validate that elesclomol selectively kills cisplatin resistant lung cancer cells through increasing ROS.

### 2.4. The effect of Elesclomol on Antioxidant System

**Figure 9:**
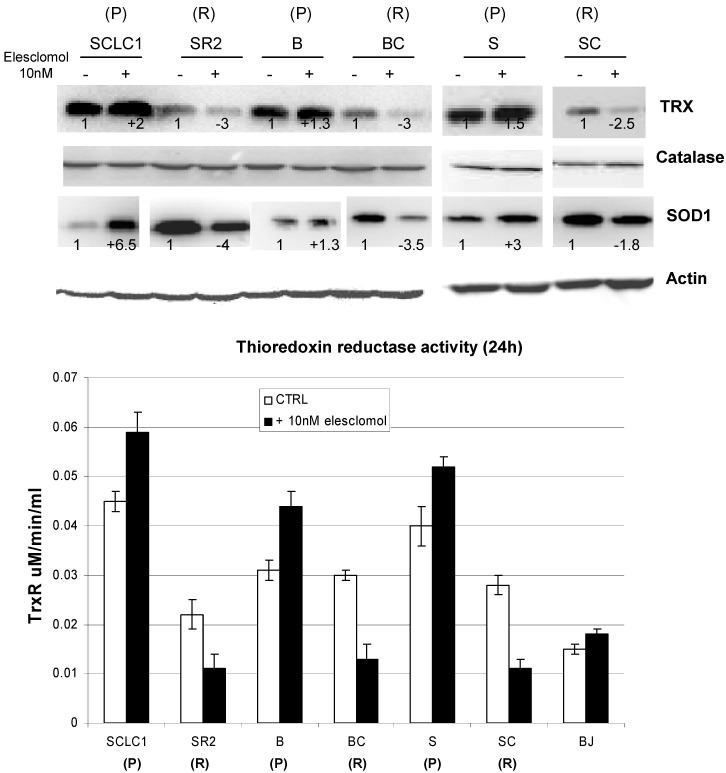
Immunoblot of lung cancer cell lines before and after treated with 10 nM of elesclomol for 24 h. TRX and TrxR activity are decreased in resistant lines and further decreased when treated with elesclomol. There is no significant change in catalase level. On the other hand, SOD1 levels are increased in all of the resistant cell lines (R) and is decreased upon treated with elesclomol. The number below each lane depicted the changes from its control which was arbitrarily set at 1. All the band intensity was normalized with actin (average p < 0.01; parental (P) *vs.* resistant (R)) (mean SD of three experiments).

It has been previously reported by several laboratories that cisplatin as well as other alkylating agents inhibit TrxR [1,7,8]. This inhibition results in lowering TRX levels and thereby increased ROS levels leading to further DNA damage and cell death. Therefore, we investigated the levels of TRX/TrxR before and after treatment with elesclomol. Interestingly, the TRX/TrxR is lower in all cisplatin resistant cell lines and further reduced after treatment with elesclomol (Figure 9). In contrast, the basal TRX/TrxR levels in parental cells are higher and further increased after elesclomol. The question remains why TRX/TrxR is lower in cisplatin resistant cells and further decreased after elesclomol. It is possible that repeated exposure to cisplatin results in lower TRX due to the fact that cisplatin is known to target TrxR. Thus, decreasing the amount of TRX while increasing the amount of other antioxidant system(s) may be a means to evade the cytotoxic effect of cisplatin.

Why elesclomol further decreases TRX activity is not known; however, the answer may lie with the level of SOD1, since SOD1 is also attenuated after elesclomol treatment (to be discussed later).

**Figure 10:**
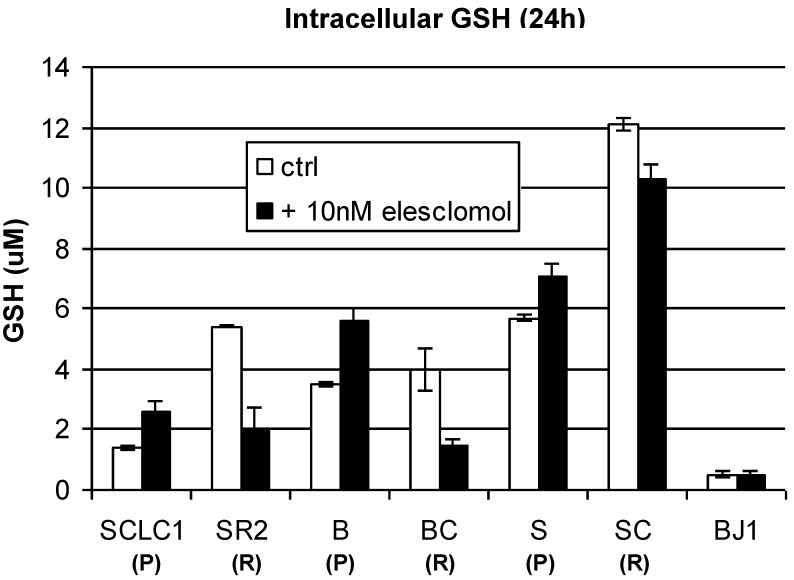
Total cellular of GSH in lung cancer cell lines treated with 10 nM of elesclomol for 24 h. Note: After treatment with elesclomol, the levels of GSH are decreased in all of the resistant cell lines (R) (Mean SD of three experiments).

We then investigated the other two important antioxidant systems, catalase and glutathione (GSH) levels, before and after exposure to elesclomol. As shown in Figure 9 and Figure 10, the basal levels of catalase are similar in the parental and resistant cells and showed no significant change upon exposure to elesclomol. However, we have found that the basal levels of GSH are higher in cisplatin resistant cells, but upon exposure to elesclomol, the levels decreased. Conversely, although the basal levels of GSH are lower in parental cells, these cells are able to activate GSH system after exposure to elesclomol (Figure 10). Overall, our data suggest that parental cells are able to activate the antioxidant system readily upon exposure to oxidative stress induced by elesclomol and are thereby able to evade apoptosis at doses that kill resistant cells. It is not clear why cisplatin resistant cells can not increase intracellular GSH under elesclomol treatment. It is possible that cisplatin resistant cells may already express maximal levels of GSH in order to compensate for high basal ROS level and are unable to increase these levels any further.

Another important factor that may explain high basal levels of ROS in cisplatin resistant cells and its collateral sensitivity to elesclomol is superoxide dismutase (SOD). Consequently, we investigated SOD1 in this panel of cisplatin sensitive and resistant cells, both before and after elesclomol treatment. SOD is a metalloenzyme that is important to capture superoxide (O_2_^−^) and convert O_2_^−^ into H_2_O_2_ and oxygen. H_2_O_2_ is then detoxified by TRX, catalase and/or GSH. Thus, the amount of SOD1 present is crucial for the cellular antioxidant defense mechanism. Figure 9 shows that the basal levels of SOD1 are much higher in cisplatin resistant cell lines; however, upon exposure to elesclomol, the levels of SOD1 increased only in parental cells but decreased in cisplatin resistant cells. In contrast, when compared to parental cell lines, TRX/TrxR is lower in resistant cells and the levels further reduced when treated with elesclomol (Figure 9).

We have already shown that cisplatin resistant cells have high basal levels of ROS/O_2_^−^. Thus, they require high levels of SOD1 to capture ROS/O_2_^−^. However, upon exposure to elesclomol, SOD1 is reduced, but ROS and H_2_O_2_ continue to rise which reflects high levels of oxidative stress. The underlying mechanism(s) for this is not clear. It is possible that cisplatin resistant cells may not be able to effectively remove H_2_O_2_ due to inability to increase TRX and GSH expression. In order to avoid the cytotoxic effect of excessive intracellular H_2_O_2_, the cisplatin resistant cells will generate less intracellular SOD1 in an attempt to decrease H_2_O_2_ formation upon exposure to elesclomol. However, despite decreasing SOD1, the amount of H_2_O_2_ generated is still too excessive and ultimately lead to cell death. In contrast, parental cells can turn on SOD1 readily to capture ROS/O_2_^−^ and the resulting H_2_O_2_ is rapidly detoxified by the antioxidant system upon exposure to elesclomol.

## 3. Experimental Section

### 3.1. Cell Lines

SCLC1&SCLCSR2, SCLCB&SCLCBC, NSCLCS&NSCLCSC have been previously characterized [9,10,11,12]. NSCLCF was derived from metastatic adenocarcinoma of the lung to the brain. The NSCLCFC line was established from NSCLCF by repeated exposure to cisplatin. Cell line LE was established from the pleural fluid of a patient who failed cisplatin. An immortalized normal fibroblast cell line (BJ-1) was obtained from Clontech (Mountain View, CA, USA). Note: SCLCSR2 exhibits 20 fold resistances to cisplatin, NSCLCSC exhibits 7-fold resistance to both cisplatin and carboplatin. SCLCBC exhibits 10-fold resistance to cisplatin and carboplatin, NSCLCFC exhibits 8-fold resistance to cisplatin and carboplatin.

### 3.2. Compounds and Antibodies

*N*′^1^,*N*′^3^-Dimethyl-*N*′^1^,*N*′^3^-bis(phenylcarbonothioyl)propanedihydrazide (elesclomol) was kindly provided by Synta Pharmaceuticals (Lexington, MA, USA). Antibodies for Caspase-3 and SOD1 were purchased from Cell Signaling Inc. (Danvers, MA, USA). Antibodies for TRX and catalase were purchased from BD Bioscience (San Jose, CA, USA). Antibody for Actin was purchased from Sigma Chemicals (St. Louis, MO, USA).

### 3.3. Growth Inhibitory Assay

Cells were seeded in 24-well dishes and treated with various concentrations of elesclomol for 24, 48, or 72 h. The procedure was described previously [9,13]. Briefly, the culture mediums as well as the trypsinized cells were collected and this mixture was centrifuged at 400× *g* for 5 min. The supernatant was discarded, and resuspended in 1 mL of Hank's buffer and assayed for live cells using a Vi-Cell cell viability analyzer (Beckman Coulter, Inc.)

### 3.4. Apoptosis Assay

Untreated and treated cells were collected and apoptosis was analyzed with caspase fluorescein-conjugated V-D-FMK (R&D Systems, Minneapolis, MN, USA). The cell pellet was collected and suspended in 100 µL of staining solution (10 µL of fluorescein and 20 µL of propidium iodide (PI) in 1 mL of PBS). The suspension was incubated at 37 °C for 30 min, washed with PBS, centrifuged to remove unbound reagent and resuspended in 500 µL of PBS. Samples were analyzed using a Coulter XL Flow Cytometer.

### 3.5. Assay for Intracellular ROS and H_2_O_2_

Untreated and treated cells were collected and intracellular ROS or H_2_O_2_ were measured by incubating with 20 µM of CM-H_2_DCF-DA (Invitrogen, Carlsbad, CA, USA) or with 10 µM of acetyl-pentafluorobenzenesulfonyl fluorescein (APFB) (EMD, La Jolla, CA, USA), respectively, at 37 °C for 30 min in the dark. Then the cells were washed once with PBS and centrifuged to remove impermeable reagents. Cells were resuspended in 500 µL of PBS and analyzed in a Coulter XL Flow Cytometry unit (excitation at 495 nm and emission at 529 nm).

### 3.6. Assay of Thioredoxin Reductase (TrxR)

A TrxR kit (Cayman Chemical, Ann Arbor, MI, USA) was used to measured total cellular thioredoxin reductase. Cells were seeded at 4 × 10^5^ and cell lysate was prepared by sonication using the conditions recommended by the manufacturer. Total TrxR activity was detected by measuring the reduction of DTNB with NADPH to TNB by UV spectrophotometer at 405 nm. The cellular TrxR contents were calculated using the standard curve generated in parallel experiments. Protein concentrations of each condition were verified using the Micro BCA Protein Assay (Thermo Scientific, Rockford, IL, USA).

### 3.7. Assay of Glutathione (GSH)

A GSH assay kit (Cayman Chemical, Ann Arbor, MI, USA) was used to measure total cellular glutathione. Cells were seeded at 4 × 10^5^ and cell lysate was prepared by sonication and deproteination using the conditions recommended by the manufacturer. Total GSH was detected by measuring the product of glutathionylated DTNB by UV spectrophotometer at 405 nm. The cellular GSH contents were calculated using the standard curve generated in parallel experiments.

### 3.8. Western Blot Analysis

Cells were seed at 1 × 10^5^/ml onto 100 mm dishes, treated, collected, lysed and immunoblotted with indicated antibody. Detail procedure was described in our previous publications [9,13]. Briefly, cell lysis was completed by sonication and centrifugation. The total protein was separated on an SDS-PAGE, transferred onto a nitrocellulose membrane (Amersham Biosciences, Piscataway, NJ) and immunoblotted with the indicated antibodies. Bands were measured using a molecular imager Chemidoc system with Quality One software (Bio-Rad, Richmond, CA).

## 4. Conclusions

**Scheme 1:**
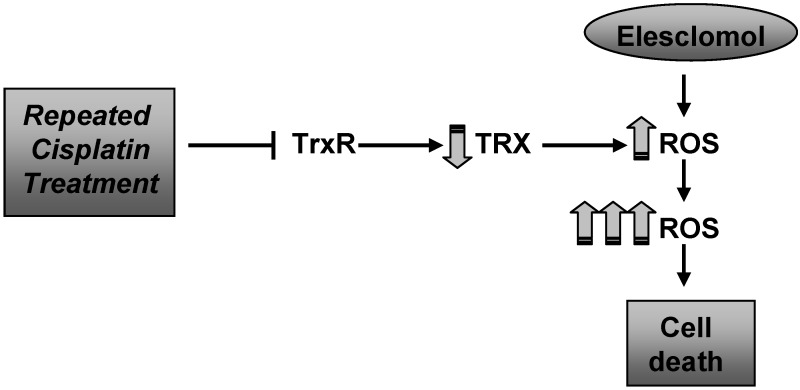
Repeat exposure to cisplatin elevates ROS production in lung cancer. The use of elesclomol to further increase ROS can push cisplatin resistant cells beyond the threshold of tolerability and hence create a potential therapeutic window.

In this report, we have presented a novel alternative approach to selectively kill cisplatin resistant cells. Cisplatin is known to inhibit TrxR which leads to elevated ROS levels and repeated exposure to cisplatin leads to lower TrxR/TRX levels and high basal ROS levels (see Scheme 1). Cisplatin resistant cells may adapt to survive under high basal levels of ROS by activating other antioxidant systems to compensate. Exposure to elesclomol leads to further increase in ROS levels and results in cell death while normal and parental cells which have lower levels of ROS can survive at the same dosage of elesclomol.

Targeting cancer cells by ROS mediated mechanisms was initially reported by Trachootham *et al*. as an effective strategy to treat cancer cells that possess unique redox mechanisms [14]. This concept has been shown to effectively kill fludarabine resistant CLL and imitanib resistant CML cells that have high basal ROS levels [15]. The investigators used PEITC which interferes with GSH as a mean to increase ROS. There are multiple means of increasing ROS, either by direct generation of ROS or interfering with the antioxidant system. The efficacy of each method is greatly dependent on both the active redox system of the tumor cells and the cause of increasing ROS.

In our cisplatin resistant lung cancer cell lines, we have found that elesclomol is very effective in generating ROS and selectively killing them. This finding can be translated into future clinical trial to treat patients who fail cisplatin.
